# Evaluation of Diaphragm Position Variations During Proton Therapy for Pediatric Patients With Neuroblastoma

**DOI:** 10.7759/cureus.58317

**Published:** 2024-04-15

**Authors:** Takahiro Kato, Yuki Narita, Kimihiro Takemasa, Masaki Suzuki, Katsuji Yokota, Hisashi Yamaguchi, Masao Murakami

**Affiliations:** 1 Department of Radiation Physics and Technology, Southern Tohoku Proton Therapy Center, Koriyama, JPN; 2 Department of Radiological Sciences, School of Health Sciences, Fukushima Medical University, Fukushima, JPN; 3 Department of Radiation Oncology, Southern Tohoku Proton Therapy Center, Koriyama, JPN

**Keywords:** external radiation therapy, interfractional motion, intrafractional motion, diaphragm motion, neuroblastoma, pediatric patient

## Abstract

Background

To evaluate the respiratory-induced intrafractional diaphragm motion and interfractional diaphragm displacement in pediatric patients with neuroblastoma (NBL).

Materials and methods

Ten pediatric patients with a mean age of 4.5 years (range: 1.8-8.7 years) with abdominal NBL treated with proton therapy (PT) have been evaluated. Intrafractional motion and interfractional displacement have been analyzed by using cine radiography and orthogonal X-ray images, respectively. In each case, the cranio-caudal positions of the diaphragm have been measured as an index. This study has investigated the possible correlations between intrafractional diaphragm motion and height. Additionally, interfractional displacement and its time trend during the treatment course have been analyzed.

Results

The average right and left diaphragm intrafractional motions of 8.3 mm (range: 4.4-11.5 mm) and 6.4 mm (range: 2.2-11.8 mm) were observed, respectively; however, no significant correlation has been observed with height. An interfractional displacement of 5 mm or more has been observed in 20 out of 152 fractions (13%). The average absolute value of the interfractional displacement was 2.5 mm (range: 0-8.6 mm). Interfractional displacement did not show a peculiar tendency throughout the treatment period.

Conclusions

It was suggested that respiratory-induced diaphragm position variation in children varies greatly among individuals, and accurately estimating it based on height is difficult. Thus, these individual evaluations are considered indispensable.

## Introduction

Neuroblastoma (NBL) is the most common type of abdominal cancer in children. Approximately 65% of the site of occurrence is the abdomen, half of which is the adrenal medulla, and the rest is known to occur from the neck, chest, pelvis, etc. [1]. Treatment strategies are generally determined according to the risk classification of the Children’s Oncology Group. Radiation therapy to the primary tumor and regional nodes is currently delivered to patients with high-risk NBL [2]. In children, extra caution is required with the delivery of radiation due to the radiosensitive nature of their developing tissue.

Gross tumor volume (GTV) in NBL is generally referred to as the primary lesion on diagnostic images after induction chemotherapy and regional lymph node metastases found at the time of initial onset. The clinical target volume (CTV) is defined as GTV with a margin of 10 to 15 mm, with appropriate modifications considering the anatomical and pathological progression patterns. Planning target volume (PTV) is usually determined for each clinic considering the setup and internal errors. Especially in the abdomen, which is a frequent site, setting an appropriate internal margin considering respiratory motion is necessary [3]. Pediatric safety margins are often based on data from adult studies; however, adult-based margins might be too large for children. Moreover, the information on respiratory motion in children, especially for those less than five years of age, is scarce, and the problem of setting an appropriate internal margin still exists. The analysis of internal organ motion in pediatric NBL has been performed [4-13], but most reports are from a limited number of institutions, and it is hard to say that there is sufficient consensus about internal organ motion in pediatric patients. Therefore, the intrafractional diaphragm motion and interfractional diaphragm displacement in pediatric patients for NBL have been analyzed to contribute to the accumulation of evidence.

## Materials and methods

Patient background

Ten pediatric patients who had been treated by proton therapy (PT) at our institution for abdominal NBL between May 2016 and December 2020 have been included in this study. Patients were included when the diaphragm was visible on orthogonal setup images. Mediastinal surgery, causing diaphragmatic dysfunction, was an exclusion criterion because the remaining diaphragmatic motion was minimal and therefore not representative of normal free breathing. Patient characteristics are summarized in Table 1. Tumor location was determined based on whether the left or right side was dominant in terms of the proportion of primary tumor present. In most cases, the tumor was located just below the diaphragm. Our study was approved by the institutional review board of our institution. The mean age was 4.5 years (range: 1.8-8.7 years). The median height was 95 cm (range: 78-120 cm).

**Table 1:** Patient characteristics and treatment details No. of treatment fractions indicates the total number of treatment fractions. Tumor location indicates whether the tumor is located on the left or right side. M: male; F: female; SD: standard deviation.

No.	Gender	Age (years)	Height (cm)	Weight (kg)	No. of treatment fractions	Tumor location	Anesthesia
1	M	1.8	78	10	17	Left	Yes
2	M	2.5	80	11	17	Right	Yes
3	M	2.9	93	15	17	Left	Yes
4	M	3.2	87	11	17	Left	Yes
5	M	3.3	86	12	17	Left	Yes
6	M	3.8	97	15	17	Left	No
7	F	5.2	105	16	11	Left	No
8	M	5.8	111	19	11	Right	Yes
9	M	8.1	120	23	11	Right	No
10	F	8.7	119	16	17	Left	No
Mean	–	4.5	98	15	15	–	–
SD	–	2.4	15	4	3	–	–

Simulation procedure

During the simulation, the patients were placed in the supine position with both arms raised, and six patients were treated under general anesthesia during the simulation and each treatment fraction. A vacuum cushion was used to immobilize the body. Aquilion LB (Canon Medical Systems, Otawara, Japan) was used for computed tomography (CT) scans, and images were taken in 2-mm slices. Patients fasted for at least four hours before the simulation. For respiratory control, respiratory-gated scans were performed during the end-exhalation phase, by applying the respiratory monitoring system, AZ-733V (Anzai Medical, Tokyo, Japan).

The irradiation method was the wobbler method, which is one of the passive scattering methods [14]. The prescribed dose was determined as 19.8 Gy relative biological effectiveness (RBE)/11 fractions or 30.6 Gy (RBE)/17 fractions at the isocenter for the standard protocol according to the Japan Childhood Cancer Group Neuroblastoma Committee [2]. The RBE value of 1.1 was used in this study. The former and latter protocols have been applied in seven and three cases, respectively (Table 1). Hitachi’s proton-type Particle Therapy System (Hitachi, Kashiwa, Japan) and XiO-M (Hitachi, Kashiwa, Japan) have been used as the PT machine and the treatment planning system, respectively.

Measurement of intrafractional diaphragm motion

Respiratory-induced diaphragm motion in the cranio-caudal direction was measured by cine radiography using a fluoroscope. Ultimax-i DREX UI80 (Canon Medical Systems, Otawara, Japan) was used for fluoroscopy. Similarly to CT scanning, during fluoroscopic measurement, the movement of the abdominal wall was monitored using AZ-733V, and after confirming that breathing was stable, imaging of two breathing cycles was performed.

Measurement of interfractional diaphragm displacement during the course of PT

**Figure 1:**
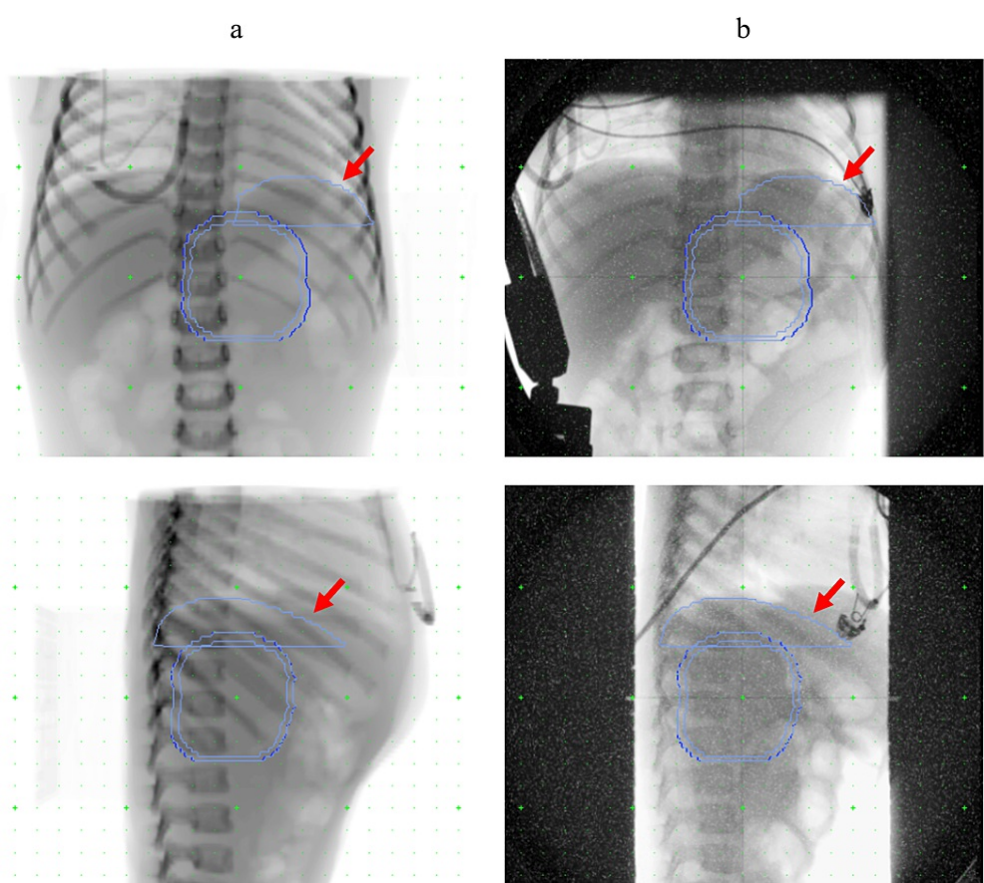
Frontal (upper) and lateral (lower) views during patient positioning (a) Digitally reconstructed radiographs obtained from the XiO-M treatment planning system (RTP). (b) X-ray images during patient setup on vertebral body matching. Red arrows indicate left diaphragm contours delineated on XiO-M RTP. Other contours in each image show the clinical target volume and planning target volume.

All conditions, including dietary restrictions at the time of each treatment, were the same as at the time of the simulation. Patient setup is performed every time immediately prior to beam irradiation using the orthogonal X-ray imaging system and the six-degrees-of-freedom couch. Our system is not equipped with an in-room CT or cone-beam CT (CBCT); therefore, the setup using the vertebral body, diaphragm, and markers as indices is performed for each case based on the orthogonal X-ray images. Figure 1 shows images of the setup in the frontal and lateral views. The contour information delineated using XiO-M is shown on the digitally reconstructed radiograph (DRR) and orthogonal setup images before treatment. The ipsilateral diaphragm was carefully delineated on the XiO-M by a single physicist and used as a reference index for positioning. The red arrows in Figure 1 indicate left diaphragm contours delineated on XiO-M. After the patient setup was performed using the vertebral body as an index, the orthogonal X-ray imaging was performed at the end of exhalation by using AZ-733V. Thereafter, the ipsilateral diaphragm position displacement on each treatment was measured using the DRR as a reference. There is almost no change in the position of the diaphragm in the left-right or anterior-posterior directions, and most of it is in the cranio-caudal direction. Therefore, the displacement was measured only in the cranio-caudal direction. The "+" and "-" signs, respectively, indicate cranio and caudal directions. Details of this method have been reported elsewhere as diaphragm matching [15].

Analysis

Respiratory-induced diaphragm motion on both sides in the cranio-caudal direction measured by cine radiography using a fluoroscope was analyzed as intrafractional motion. We analyzed the Pearson correlation between the diaphragm motion and height. The ipsilateral diaphragm position displacement for each treatment time was measured using orthogonal X-ray images as interfractional displacement. These results have been analyzed in a cumulative histogram and box plots for each patient. Additionally, a time trend analysis of these data during the course of treatment was performed.

## Results

**Figure 2:**
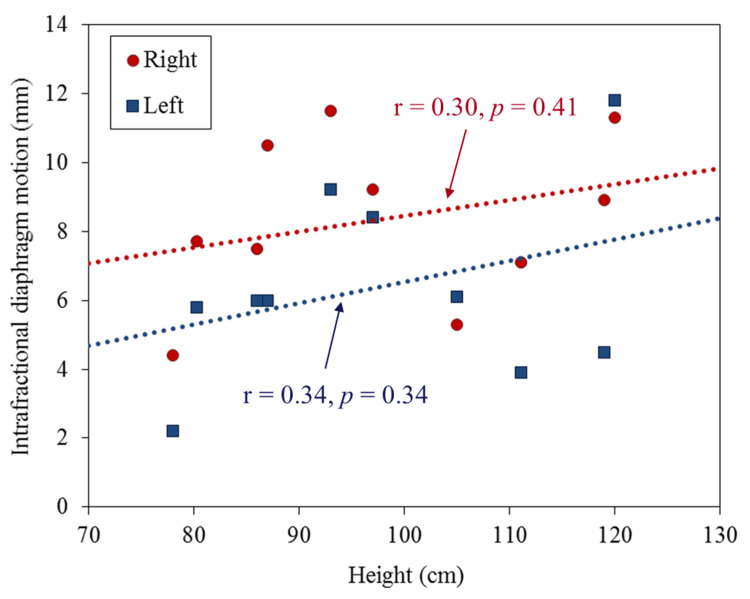
Scatter plots with regression lines describing relationships between the intrafractional diaphragm motion and height The Pearson correlation between the intrafractional diaphragm motion and height was also analyzed, where r is the linear correlation coefficient and p is the probability for no correlation. Right and left indicate the right diaphragm and the left diaphragm.

**Figure 3:**
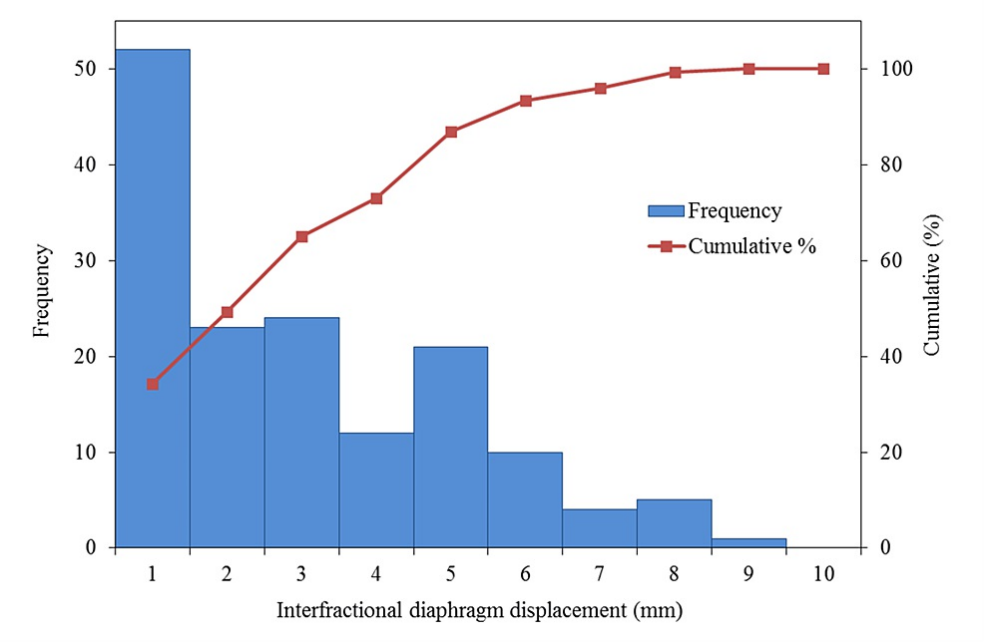
Cumulative histogram of the interfractional diaphragm displacement distribution The red line indicates the cumulative percentage.

**Figure 4:**
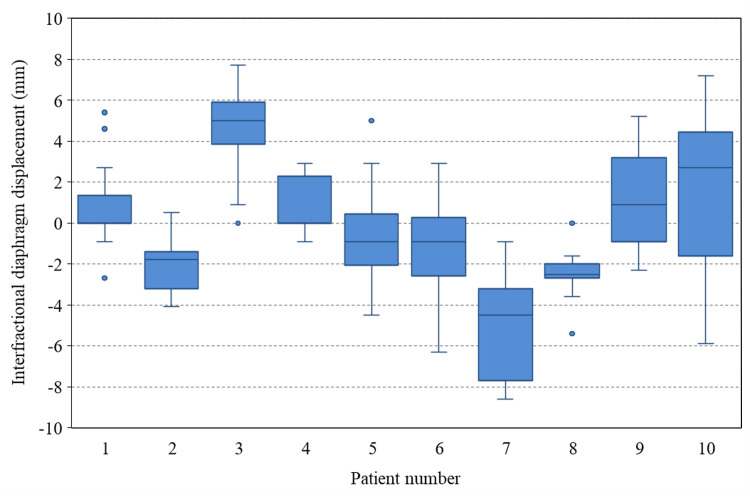
Boxplot for the interfractional diaphragm displacement Boxplot for the interfractional diaphragm displacement relative to the digitally reconstructed radiographs obtained from the XiO-M treatment planning system for all 10 patients. The zero line distinguishes the opposite directions, wherein the "+" and "–" signs indicate cranial and caudal directions, respectively.

**Figure 5:**
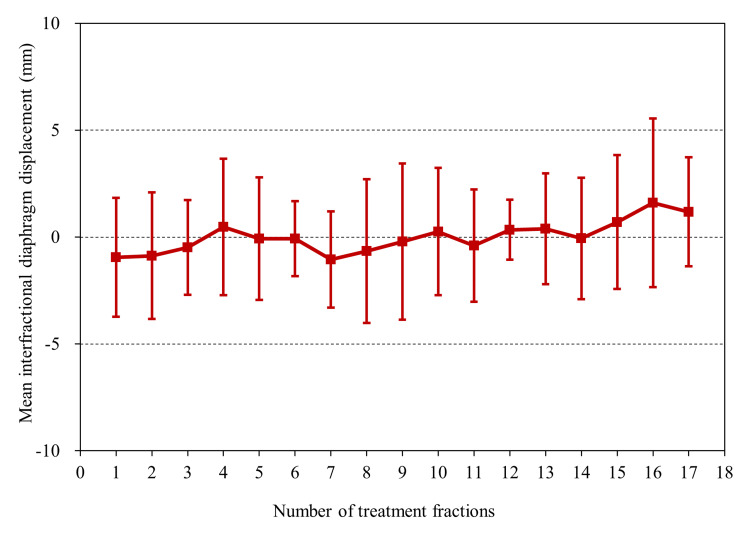
Time trend of the mean interfractional diaphragm displacement The error bars indicate one standard deviation.

Figure 2 shows a scatter plot of intrafractional motion on both sides and height. An average right and left intrafractional motion of 8.3 mm (range: 4.4-11.5 mm) and 6.4 mm (range: 2.2-11.8 mm) have been observed, respectively, but no significant correlation has been observed with height. After analyzing the average value of the right and left diaphragm motion using a t-test, results showed that the right side was significantly larger than the left side (p < 0.01). Figure 3 shows a cumulative histogram of 152 fractions in all cases of interfractional displacement. A displacement of 5 mm or more was observed in 20 out of 152 fractions (13%). The average absolute value of the interfractional displacement was 2.5 mm (range: 0-8.6 mm). The mean maximum range of the interfractional displacement in individual cases was 6.6 mm (range: 3.8-13.1). Figure 4 shows a boxplot of the interfractional displacement for each patient. There seems to be a large interpatient variation. No displacement of 5 mm or more was observed in four of 10 cases during the treatment course. Figure 5 shows the time trend analysis results of the mean interfractional displacement during the course of PT. No specific tendency was observed throughout the treatment course.

## Discussion

Several reports of pediatric respiratory-induced position variations in NBL have been presented [4-13]. However, reports were limited to four facilities; of these, only one facility reported from Asia, and it is hard to say that a global consensus has been reached regarding the setting of internal margin. Of the 11 reports on measures for pediatric respiratory-induced position variations mentioned above [4-13,16], only three reports comprehensively analyzed both intrafractional and interfractional position variations [10,13,16]. Moreover, among these reports, only seven cases were under five years old at the maximum, and the fact is that information on very small children is still lacking. Although our analysis has a small number of cases (10), this is the first report from East Asia, and it is a comprehensive analysis of intrafractional and interfractional position variations: eight out of 10 cases are under five years old. Therefore, it is considered to be valuable data to contribute to the accumulation of evidence.

We tried to analyze the correlation between the left and right intrafractional motion and height, but no correlation was found. Some reports analyzing the correlation between the diaphragm and abdominal organs and height have been presented. Pai Panandiker et al. have reported that renal cranio-caudal motion correlates with height [6]. Uh et al. have also reported that the right diaphragm motion significantly correlated with height [9]. Conversely, Huijskens et al. reported that no correlation was found between the diaphragm motion and height [10]. Guerreiro et al. similarly reported that no correlation was found between the liver, spleen, and contralateral kidney and height [16]. From these reports and our study, consensus has not always been obtained regarding the correlation between respiratory-induced position variation of abdominal organs and height, and an analysis based on a larger number of cases is considered necessary. From the results of this analysis, an interesting result was observed that the motion on the right diaphragm was significantly larger than that on the left diaphragm. This has often been pointed out in previous reports and may be a universal trend [6,11]. It is known that the incidence of NBL is 2-3 on the right-to-left side [1], which is slightly higher on the left side, but it should be understood that the internal margins required on the left and right sides may differ. Pai Panandiker et al. have reported an average of 5.1 mm (range: 3.0-10.0), although the distinction between the left and right was not specified [6]. Kannan et al. have reported 4.4 ± 2.0 mm on the right and 3.6 ± 2.3 mm on the left [8]. Uh et al. examined 35 cases and summarized the results in detail for each case [9]. When the results of cases under eight years old were extracted from their results as in our study, the right diaphragm motion averaged 4.7 mm (range: 1.2-9.0 mm). From the above, it was found that our results tended to have a slightly larger motion than the previous reports. This is thought to be due to the fact that the motion of the right diaphragm was 10 mm or more in three of the 10 cases. Moreover, two out of three cases were three years old or younger. To the best of our knowledge, there has been no report that there is a motion of 10 mm or more in less than three years old so far, and it is considered to be one of the new findings shown in this study. Uh et al. have emphasized that the significance of implementing respiratory mobility measures under eight years old may be limited [9]. However, a motion of 10 mm or more even under three years old (height: 93 cm or less) has been confirmed, suggesting that concluding from the age and height alone would be difficult.

Due to the small number of cases analyzed, detailed statistical analysis was not performed, but no findings suggesting an age-related tendency have been confirmed in Figure 4. Furthermore, it can be seen that patient 3 and patient 7 have systematic displacements over the entire course. Since displacements were observed from the beginning of treatment in these cases, CT imaging was performed again after the end of the initial treatment session, and whether or not the treatment could be continued is being discussed. As a result of careful comparison with the planning CT, it was confirmed that the diaphragm was displaced, but the CTV was not significantly displaced, and it was judged that the treatment was continued. Three out of 10 cases underwent unscheduled CT due to diaphragmatic displacement during the treatment course, but only one of them was replanned for safety reasons. Even in such cases, the analysis of interfractional displacement is evaluated based on the initial CT as a reference. Additionally, some cases had the diaphragm displaced but not the surgical clip. Therefore, it should be noted that the diaphragm displacement does not directly indicate treatment accuracy. Nazmy et al. have reported a maximum range of displacement of 11 mm in the right diaphragm [5]. Huijskens et al. have reported that the interfractional variability (i.e., the standard deviation over mean amplitudes from each fraction) was 1.4 mm [10]. As a result of the same evaluation in our study, the value was 2.1 mm, and the interfractional displacement was considered to be comparable to the previous reports. Since it has been emphasized that interfractional errors tend to be larger than intrafractional errors [16], confirming the internal reproducibility using some method during the treatment course is important.

Although a report has mentioned the time trend of setup errors in children [4], there is no report that analyzes the time trend of interfractional displacement. Therefore, this study analyzed the time trend of interfractional displacement during the treatment course. From our results, it was considered appropriate to judge that no constant tendency was observed during the course of treatment.

This study has evaluated the intrafractional motion and interfractional displacement based on the cine radiography and orthogonal X-ray images, respectively. Although these methods may be effective in evaluating the diaphragm with clear contrast and surgical clip, it is clear that it is not sufficient for evaluating soft tissues, including the kidney. MRI is often performed during treatment planning simulation, and in that case, cine MRI is also added to evaluate the motion of the soft tissue. However, when an MRI is not performed, there is no choice but to estimate the motion from information such as the diaphragm or surgical clip. The same can be said for the analysis of interfractional displacement. It has been emphasized that there are large individual differences in both intrafractional and interfractional position variations in children [10,16]. From this result, intrafractional motion of 10 mm or more was observed even under three years old, suggesting that accurately predicting the intrafractional motion based on age and height is difficult. The guidelines stipulate a minimum PTV margin of 5 mm [2]. Contrastingly, Nazmy et al. emphasized that the PTV margin of 5 mm is too tight without CBCT [5]. Since it is considered that consensus has already been obtained that the cranio-caudal motion is significantly large for both intrafractional and interfractional position variation, it is realistic to set an anisotropic margin such as 7 mm in the cranio-caudal direction as the PTV margin. Presently, our clinic sets such a margin and routinely evaluates the respiratory motion, and monitors during the treatment course as shown in this study. In the future, considering setting an individualized margin will be necessary, and for that purpose, using a four-dimensional CT during simulation and CBCT during the treatment course will be necessary. It is a prerequisite to consider the balance between image quality and radiation exposure in advance, but we would like to actively consider its use in optimized conditions.

One of the limitations of this study is that the intrafractional motion for each treatment could not be analyzed. The intrafractional motion shown in this study is a one-time result during simulation. Intrafractional motion can change from day to day [10], so even if the motion is small during simulation, it should not be overconfident. This issue is not unique to children, but it should be recognized that uncertainty is always present in the assessment of respiratory-induced motion. However, a report showed that there is relatively little variation in children [13], and we hope to systematically analyze it in the future. Another limitation is that, like previous studies [4-13], we were not able to investigate the effects of anesthesia. The presence or absence of anesthesia may affect the results of respiratory-induced diaphragm motion, but the small number of analyzed cases makes it difficult to elucidate this issue. This point may also be an ongoing issue.

## Conclusions

Intrafractional and interfractional position variations have been analyzed for 10 patients with NBL. Cases of intrafractional motion of 10 mm or more even under three years old (height: 93 cm or less) have been confirmed. Therefore, it was suggested that accurately predicting the intrafractional motion based on age and height is difficult. Additionally, although the frequency was low, the interfractional displacement of 7 mm or more was observed, suggesting the need for regular monitoring of internal errors during the treatment course.
